# Lower preoperative vitamin D levels are associated with poor clinical outcomes in elderly patients with osteoporotic vertebral compression fractures after percutaneous vertebroplasty

**DOI:** 10.3389/fmed.2026.1814433

**Published:** 2026-05-11

**Authors:** Fuzhang Wu, Yongbing He, Zhenhua Zhang, Buzhou Chen, Pengli Zhang

**Affiliations:** Department of Orthopedics, Anhui Provincial Corps Hospital, Chinese People’s Armed Police Force, Hefei, China

**Keywords:** functional recovery, osteoporotic vertebral compression fracture, pain, percutaneous vertebroplasty, vitamin D

## Abstract

**Objective:**

Vitamin D deficiency is common among elderly individuals with osteoporosis; however, its prognostic significance in patients undergoing percutaneous vertebroplasty (PVP) for osteoporotic vertebral compression fractures (OVCFs) remains unclear. This study evaluated the association between preoperative vitamin D status and postoperative pain and functional recovery following PVP.

**Methods:**

This retrospective study included 609 elderly patients with single-level OVCFs treated with PVP between January 2021 and December 2024. Preoperative serum 25-hydroxyvitamin D [25(OH)D] levels were categorized as severe deficiency (<10 ng/mL), deficiency (10–20 ng/mL), insufficiency (20–30 ng/mL), and sufficiency (>30 ng/mL). Pain and function were assessed using the Visual Analog Scale (VAS) and Oswestry Disability Index (ODI) preoperatively, on postoperative day 1, at 1 month, and at 1 year. Linear mixed-effects models (LMMs) evaluated longitudinal changes, and multivariable regression examined independent associations with 1-year outcomes.

**Results:**

Visual Analog Scale and ODI scores improved significantly over time in all groups (both *p* < 0.01). However, patients with lower vitamin D levels consistently had higher pain and disability scores at each follow-up. Significant time × group interactions were observed (both *p* < 0.01). After multivariable adjustment, lower vitamin D status remained independently associated with higher 1-year VAS (*β* = 0.293, 95% CI: 0.230, 0.356, *p* < 0.01) and higher ODI scores (β = 2.179, 95% CI: 1.854–2.503, *p* < 0.01).

**Conclusion:**

Lower preoperative vitamin D levels are independently associated with worse postoperative pain relief and functional recovery after PVP in elderly patients with OVCFs.

## Introduction

Osteoporotic vertebral compression fractures (OVCFs) are a major cause of pain, disability, and impaired quality of life among older adults. With rapid population aging, the burden of osteoporotic fractures continues to rise worldwide and has become a significant public health concern (1). A recent systematic review and meta-analysis reported that the overall prevalence of osteoporotic fractures among Chinese elderly adults increased markedly from 13.2% in 2000–2010 to 22.7% in 2012–2022, with the spine identified as one of the most frequently affected sites (2). Epidemiological studies further indicate that vertebral fractures affect up to 30% of men and women aged 50 years and older, with prevalence rising steeply with advancing age (3). Given the high risk of recurrent fractures, functional decline, and excess mortality associated with OVCFs, effective management strategies are essential to mitigate their growing clinical and socioeconomic impact.

Percutaneous vertebroplasty (PVP) has been widely adopted as a minimally invasive treatment for OVCFs and is effective in providing rapid pain relief, vertebral stabilization, and early functional recovery (4). Compared with prolonged conservative management, PVP allows earlier mobilization and improved short-term outcomes in appropriately selected patients (5). However, despite technically successful procedures, postoperative outcomes remain heterogeneous. While many patients experience substantial symptom improvement, others continue to suffer from persistent pain or limited functional recovery (6). This variability suggests that factors beyond procedural technique—particularly patient-related and metabolic conditions—may play an important role in determining prognosis after PVP.

Vitamin D is a key regulator of bone metabolism, muscle strength, and neuromuscular function (7). Vitamin D deficiency is highly prevalent in elderly individuals and patients with osteoporosis and has been associated with reduced bone quality, impaired fracture healing, chronic musculoskeletal pain, and increased risk of falls (8, 9). Although emerging evidence suggests that low vitamin D status may adversely influence recovery after orthopedic interventions (10), its prognostic significance in patients undergoing PVP for OVCFs has not been well established.

Therefore, this study aimed to investigate the association between preoperative serum 25-hydroxyvitamin D [25(OH)D] levels and postoperative pain and functional outcomes in elderly patients with single-level OVCFs treated with PVP. We hypothesized that severe vitamin D deficiency is independently associated with poorer clinical outcomes after PVP, and that identifying this modifiable risk factor may help optimize perioperative management and improve postoperative recovery in this vulnerable population.

## Materials and methods

### Study design and population

This retrospective observational study included consecutive elderly patients diagnosed with OVCFs who underwent PVP at the Department of Orthopedics, Anhui Provincial Corps Hospital, Chinese People’s Armed Police Force, between January 2021 and December 2024.

The study was conducted in accordance with the Declaration of Helsinki and was approved by the Ethics Committee of Anhui Provincial Corps Hospital (approval no. 2025041). The requirement for informed consent by the Ethics Committee of Anhui Provincial Corps Hospital was waived due to the retrospective nature of the study.

Patients were eligible for inclusion if they met the following criteria: (1) age ≥65 years; (2) diagnosis of single-level OVCF confirmed by radiography and magnetic resonance imaging; (3) low-energy or atraumatic fracture; (4) treatment with unilateral or bilateral PVP; and (5) availability of preoperative serum 25(OH)D measurements.

Exclusion criteria were: (1) pathological fractures caused by malignancy or infection; (2) high-energy traumatic fractures; (3) previous spinal surgery at the involved vertebral level; (4) concomitant neurological deficits requiring decompression surgery; (5) long-term use of vitamin D supplementation prior to admission; and (6) incomplete clinical or follow-up data.

### Assessment of vitamin D status

Preoperative vitamin D measurement was routinely performed as part of the clinical assessment for elderly patients diagnosed with OVCFs who underwent PVP in our institution. Fasting venous blood samples were collected prior to surgery. Serum 25(OH)D levels were measured using a standardized chemiluminescence immunoassay. Based on commonly accepted clinical thresholds, patients were categorized into four groups: severe deficiency (<10 ng/mL), deficiency (10–20 ng/mL), insufficiency (20–30 ng/mL), and sufficiency (>30 ng/mL).

### Percutaneous vertebroplasty procedure

PVP was performed under sterile conditions and continuous C-arm fluoroscopic guidance. Patients were positioned prone with the abdomen suspended to facilitate postural reduction. The fractured vertebra was identified under fluoroscopy, and a unilateral or bilateral transpedicular approach was used according to fracture characteristics.

Following local anesthesia, a vertebroplasty needle was advanced through the pedicle into the fractured vertebral body under real-time fluoroscopic monitoring. Polymethylmethacrylate bone cement was prepared according to the manufacturer’s instructions and injected slowly during its doughy phase. Cement distribution was continuously monitored, and injection was terminated once adequate filling was achieved or if cement approached the vertebral cortex or leakage was observed. Postoperatively, patients were encouraged to ambulate early and received standard anti-osteoporotic therapy.

### Clinical outcomes

Pain intensity was evaluated using the Visual Analog Scale (VAS), and functional status was assessed using the Oswestry Disability Index (ODI). Outcomes were conducted 1 day before the operation, on the first day postoperatively, at 1-month postoperatively, and 1-year postoperatively.

Baseline demographic and clinical variables were collected from electronic medical records, including age, sex, body mass index (BMI), smoking status, bone mineral density (BMD), fractured vertebrae, comorbidities (diabetes, hypertension and cardiovascular diseases), and postoperative exercise and osteoporosis treatment. Exercise was defined as aerobic exercise, including brisk walking, jogging, dancing, swimming, and climbing, that increased the heart rate for at least 30 min and was performed for a minimum of four times a week. Data on exercise frequency and intensity were self-reported by patients. Medications for osteoporosis treatment included calcitonin and bisphosphonates. According to the treatment plan prescribed by the doctor at discharge, during the review period, medication was to be taken regularly according to the treatment course (including calcitonin and bisphosphonates used in our hospital).

### Statistical analysis

Continuous variables are presented as mean ± standard deviation or median (interquartile range), as appropriate, and categorical variables as frequencies and percentages. Comparisons among vitamin D groups were performed using one-way analysis of variance or the Kruskal–Wallis test for continuous variables and the chi-square test for categorical variables.

Because VAS and ODI scores were assessed at multiple time points (preoperatively, postoperative day 1, 1 month, and 1 year), linear mixed-effects models (LMMs) were used as the primary analysis to evaluate longitudinal changes and group differences. In these models, random intercepts were included for each patient to account for repeated measurements, and fixed effects included time, vitamin D status, and their interaction.

Multivariate linear regression analyses were conducted to evaluate the independent association between vitamin D status and VAS and ODI scores at 1 year, adjusting for potential confounders. Regression coefficients (*β*) with 95% confidence intervals (CIs) were reported. Vitamin D status was entered into the regression model as an ordinal variable (1 = sufficiency, 2 = insufficiency, 3 = deficiency, and 4 = severe deficiency), with higher values indicating worse vitamin D status.

Patients with missing preoperative vitamin D measurements or incomplete follow-up were excluded from the analysis; no imputation was performed for missing data, as the proportion of missing data was low and linear mixed-effects models (LMMs) are robust to missing-at-random (MAR) assumptions.

A two-sided *p*-value <0.05 was considered statistically significant. All statistical analyses were performed using SPSS software (version 25.0; IBM Corp., Armonk, NY, United States).

## Results

A total of 787 patients underwent PVP between January 2021 and December 2024. Among them, 178 patients were excluded: 51 with missing preoperative vitamin D measurements, 28 with pathological fractures, 15 with high-energy fractures, 20 with prior spinal surgery, 12 with neurological deficits requiring decompression, and 52 with incomplete follow-up data. The remaining 609 patients were included in the analysis.

All eligible patients were included and stratified into four groups according to preoperative 25(OH)D status: sufficiency (*n* = 151), insufficiency (*n* = 192), deficiency (*n* = 166), and severe deficiency (*n* = 100). The baseline demographic and clinical characteristics are summarized in Table 1. As shown in Table 1, baseline demographic characteristics including age, sex, body mass index, and smoking status were comparable among the four groups. The prevalence of diabetes, hypertension, fractured vertebral levels, postoperative exercise, and postoperative osteoporosis treatment also did not differ significantly. Preoperative VAS and ODI scores were comparable among the four groups (both *p* > 0.05). However, preoperative bone mineral density progressively decreased with worsening vitamin D status (*p* < 0.01). In addition, patients with lower vitamin D levels had a higher prevalence of cardiovascular diseases (*p* < 0.01).

**Table 1:** Comparison of demographic and clinical characteristics among four groups stratified by 25(OH)D status.

Variables	Vitamin D status				*P* value
	Sufficiency (>30 ng/mL)	Insufficiency (20–30 ng/mL)	Deficiency (10–20 ng/mL)	Severe deficiency (<10 ng/mL)	
*N*	151	192	166	100	
Age, yrs	69.4 ± 3.0	69.1 ± 3.1	68.8 ± 3.5	68.9 ± 3.1	0.26
Gender, *n* (%)					0.32
Male	30 (19.9%)	44 (22.9%)	38 (22.9%)	30 (30.0%)	
Female	121 (80.1%)	148 (77.1%)	128 (77.1%)	70 (70.0%)	
BMI, kg/m^2^	24.3 ± 1.4	24.6 ± 1.2	24.4 ± 1.2	24.3 ± 1.2	0.10
Smoker, *n* (%)	26 (17.2%)	35 (18.2%)	30 (18.1%)	14 (14.0%)	0.81
Preoperative BMD	−2.8 ± 0.3	−3.0 ± 0.3	−3.2 ± 0.3	−3.4 ± 0.3	<0.01
Comorbidities, *n* (%)					
Diabetes	25 (16.6%)	29 (15.1%)	36 (21.7%)	23 (23.0%)	0.23
Hypertension	28 (18.5%)	45 (23.4%)	45 (27.1%)	31 (31.0%)	0.12
Cardiovascular diseases	28 (18.5%)	48 (25.0%)	56 (33.7%)	38 (38.0%)	<0.01
Fractured vertebrae					0.79
T11	17 (11.3%)	29 (15.1%)	24 (14.5%)	13 (13.0%)	
T12	52 (34.4%)	51 (26.6%)	51 (30.7%)	34 (34.0%)	
L1	62 (41.1%)	76 (39.6%)	62 (37.3%)	39 (39.0%)	
L2	20 (13.2%)	36 (18.8%)	29 (17.5%)	14 (14.0%)	
Preoperative VAS	7.4 ± 1.5	7.5 ± 1.6	7.3 ± 1.5	7.6 ± 1.5	0.63
Preoperative ODI	68.1 ± 7.9	68.2 ± 7.2	67.0 ± 8.0	67.9 ± 7.7	0.42
Postoperative exercise, n (%)	97 (64.2%)	119 (62.0%)	94 (56.6%)	56 (56.0%)	0.41
Postoperative osteoporosis treatment, n (%)	59 (39.1%)	61 (31.8%)	68 (41.0%)	37 (37.0%)	0.30

**Figure 1:**
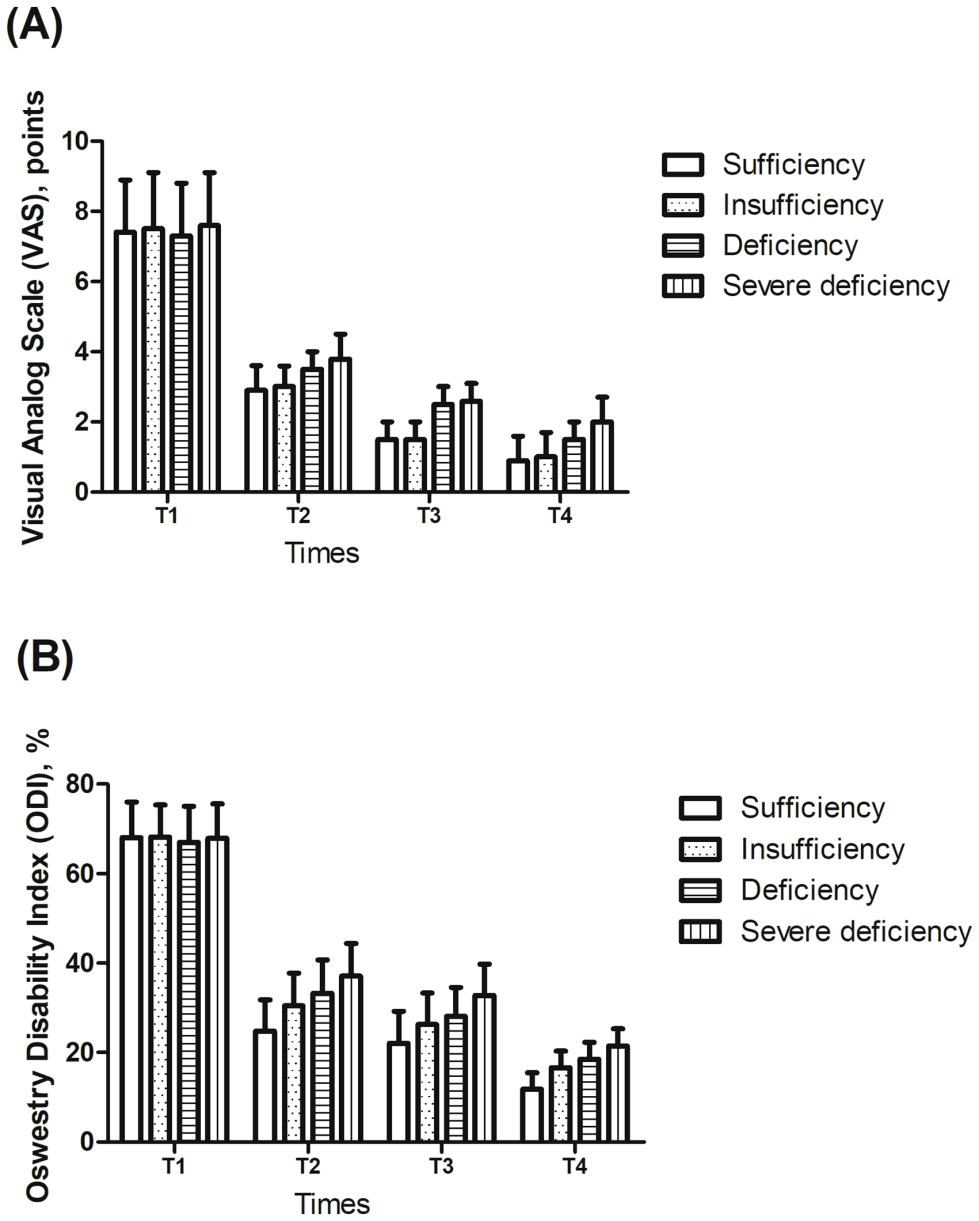
Comparison of **(A)** visual analog scale (VAS) scores and **(B)** Oswestry disability index (ODI) scores between patients stratified by 25(OH)D status at four time points: 1 day before surgery (T1), day 1 postoperatively (T2), 1 month postoperatively (T3), and 1 year postoperatively (T4). Exact values are provided in Supplementary Table S1. Linear mixed-effects models showed significant main effects of time and vitamin D status, as well as a significant time × vitamin D status interaction (all *p* < 0.01). Detailed LMM results for VAS and ODI are provided in Supplementary Tables S2, S3.

Figure 1 shows the comparison of VAS scores and ODI scores between patients stratified by vitamin D status at four time points: 1 day before surgery (T1), postoperative day 1 (T2), 1 month postoperatively (T3), and 1 year postoperatively (T4).

The exact values at all time points are provided in Supplementary Table S1. Longitudinal changes in VAS and ODI scores were analyzed using linear mixed-effects models. There was a significant main effect of time for both VAS and ODI (*p* < 0.01), indicating overall postoperative improvement across all vitamin D groups. Vitamin D status also had a significant main effect (*p* < 0.01), with patients in lower vitamin D categories exhibiting consistently higher pain and disability scores. Furthermore, the time × vitamin D status interaction was significant (*p* < 0.01), suggesting that the magnitude of improvement over time differed according to preoperative vitamin D levels. The statistical analysis results in the linear mixed-effects model for ODI and VAS scores are provided in Supplementary Tables S2, S3.

Multivariable linear regression analyses were performed to further evaluate the independent association between vitamin D status and 1-year outcomes (Table 2). After adjusting for age, sex, body mass index, smoking status, comorbidities, fractured vertebral level, postoperative exercise, and anti-osteoporosis treatment, vitamin D status remained significantly associated with both VAS and ODI scores at 1 year.

**Table 2:** Multivariate linear regression to explore the association of vitamin D status with Visual Analog Scale (VAS) and Oswestry Disability Index (ODI).

Variables	Visual Analog Scale (VAS)			Oswestry Disability Index (ODI)		
	*β*	95% CI	*P*-value	*β*	95% CI	*P*-value
Vitamin D status	0.293	0.230, 0.356	<0.01	2.179	1.854–2.503	<0.01
Age	−0.001	−0.018, 0.016	0.92	−0.009	−0.098, 0.080	0.84
Female	−0.049	−0.176, 0.079	0.45	−0.192	−0.851, 0.467	0.57
BMI	0.003	−0.040, 0.045	0.90	−0.148	−0.368, 0.072	0.19
Smoker	0.023	−0.120, 0.166	0.75	−0.391	−1.127, 0.346	0.30
Preoperative BMD	−0.296	−0.456, −0.136	<0.01	−4.88	−5.706, −4.056	<0.01
Diabetes	0.025	−0.114, 0.163	0.73	−0.327	−1.042, 0.389	0.37
Hypertension	0.012	−0.114, 0.138	0.85	−0.222	−0.874, 0.430	0.50
Cardiovascular diseases	−0.015	−0.136, 0.107	0.81	−0.531	−1.157, 0.094	0.10
Fractured vertebrae	0.004	−0.055, 0.063	0.89	0.039	−0.266, 0.345	0.80
Postoperative exercise	−0.034	−0.145, 0.077	0.55	0.214	−0.356, 0.785	0.46
Postoperative osteoporosis treatment	0.055	−0.056, 0.167	0.33	0.330	−0.247, 0.907	0.26

25(OH)D status was treated as an ordinal variable (1 = sufficiency, 2 = insufficiency, 3 = deficiency, 4 = severe deficiency); higher values indicate worse vitamin D status.

Specifically, worsening vitamin D status was independently associated with higher VAS (*β* = 0.293, 95% CI: 0.230, 0.356, *p* < 0.01) and higher ODI scores (*β* = 2.179, 95% CI: 1.854–2.503, *p* < 0.01). Preoperative BMD was also independently associated with both outcomes, with lower BMD associated with higher VAS and ODI scores, consistent with its role as a marker of osteoporosis severity.

## Discussion

In this retrospective study of elderly patients with OVCFs undergoing PVP, we found that lower preoperative vitamin D levels were significantly associated with worse postoperative pain relief and functional recovery. Although all patients experienced substantial improvement in VAS and ODI scores after surgery, those with lower vitamin D levels consistently demonstrated higher pain and disability scores during follow-up. Importantly, after adjustment for potential confounders, vitamin D status remained independently associated with 1-year clinical outcomes.

Our findings are consistent with previous studies reporting that vitamin D deficiency is highly prevalent among elderly individuals with osteoporosis and vertebral fractures and is associated with reduced bone mineral density, increased fracture risk, and impaired physical performance (11). Prior orthopedic research has also suggested that low vitamin D levels may be related to delayed fracture healing, persistent musculoskeletal pain, and inferior postoperative recovery (9). To the best of our knowledge, this is the first study to specifically evaluate the prognostic significance of preoperative vitamin D status in elderly patients undergoing PVP for OVCFs. By stratifying patients into four clinically relevant categories, we demonstrated a graded association between worsening vitamin D status and poorer 1-year outcomes, even after multivariable adjustment. These findings further highlight the potential prognostic importance of vitamin D status in this clinical setting.

Several biological mechanisms may underlie this association. Vitamin D plays a central role in calcium homeostasis, bone mineralization, and musculoskeletal function. Deficiency has been linked to impaired bone remodeling, deterioration of trabecular microarchitecture, reduced muscle strength, and chronic musculoskeletal pain (12). In our cohort, lower vitamin D levels were also associated with reduced preoperative BMD, supporting the biological plausibility of our findings. In addition, vitamin D receptors are expressed in skeletal muscle, and inadequate vitamin D status may compromise paraspinal muscle function, thereby limiting functional recovery after vertebral stabilization (13). Emerging evidence also suggests that vitamin D may influence inflammatory pathways and pain perception, which could further contribute to persistent postoperative pain (14).

From a clinical perspective, our results underscore the potential value of routine vitamin D assessment in elderly patients with OVCFs. While PVP effectively restores vertebral stability and provides rapid pain relief, metabolic factors such as vitamin D status may influence long-term recovery. Identification of patients with lower vitamin D levels may help clinicians recognize individuals at higher risk of suboptimal outcomes. Whether perioperative vitamin D optimization can improve postoperative recovery warrants confirmation in prospective interventional studies.

Several limitations should be considered when interpreting our findings. First, vitamin D levels were assessed only preoperatively, and a single measurement may not accurately reflect long-term status; seasonal variation and postoperative supplementation could also influence serum levels. Second, although we adjusted for multiple confounders, residual confounding from unmeasured factors such as frailty, nutritional status, and fracture severity cannot be excluded. Third, patients with missing preoperative vitamin D measurements or incomplete follow-up were excluded, which may introduce selection bias, although the proportion of excluded patients was moderate. Furthermore, our findings are derived from a single center in China and may not be directly generalizable to other populations with different demographic characteristics, healthcare systems, or vitamin D distributions. External validation in multicenter cohorts is warranted. Fourth, while lower preoperative vitamin D levels were statistically associated with higher 1-year VAS and ODI scores, the magnitude of these associations was relatively modest. Specifically, the observed *β* of 0.293 for VAS is below the commonly reported minimal clinically important difference (MCID) of 1–2 points, and the *β* of 2.179 for ODI is smaller than the MCID of approximately 10 points, suggesting that the clinical impact on individual patients may be limited. Nevertheless, identifying patients with low vitamin D levels may still be valuable for perioperative risk stratification and targeted interventions in this vulnerable population. Future prospective studies are warranted to determine whether correcting vitamin D deficiency can meaningfully improve postoperative recovery.

## Conclusion

In conclusion, lower preoperative vitamin D levels were independently associated with worse pain relief and functional recovery after PVP in elderly patients with OVCFs. A graded relationship was observed between worsening vitamin D status and poorer 1-year outcomes. Assessment of vitamin D status may aid in perioperative risk stratification, and future prospective studies are needed to determine whether vitamin D optimization can improve clinical outcomes in this population.

## Data Availability

The raw data supporting the conclusions of this article will be made available by the authors, without undue reservation.
