# Supplementary material for: Lower preoperative vitamin D levels are associated with poor clinical outcomes in elderly patients with osteoporotic vertebral compression fractures after percutaneous vertebroplasty
Source: Front Med (Lausanne). 2026 May 11;13:1814433. doi: 10.3389/fmed.2026.1814433 (PMC13199125; doi:10.3389/fmed.2026.1814433)
Supplement: Supplementary file 1 [file Table_1.docx]

**Supplementary Table S1. VAS and ODI scores at all time points for each Vitamin D group**

|  | **Vitamin D status** | | | |
| --- | --- | --- | --- | --- |
|  | Sufficiency  (>30 ng/mL) | Insufficiency  (20–30 ng/mL) | Deficiency  (10–20 ng/mL) | Severe deficiency  (<10 ng/mL) |
| Visual Analog Scale (VAS) |  |  |  |  |
| 1 day before surgery (T1) | 7.4±1.5 | 7.5±1.6 | 7.3±1.5 | 7.6±1.5 |
| 1 day postoperatively (T2) | 2.9±0.7 | 3.0±0.6 | 3.5±0.5 | 3.8±0.7 |
| 1 month postoperatively (T3) | 1.5±0.5 | 1.5±0.5 | 2.5±0.5 | 2.6±0.5 |
| 1 year postoperatively (T4) | 0.9±0.7 | 1.0±0.7 | 1.5±0.5 | 2.0±0.7 |
| Oswestry Disability Index (ODI) |  |  |  |  |
| 1 day before surgery (T1) | 68.1±7.9 | 68.2±7.2 | 67.0±8.0 | 67.9±7.7 |
| 1 day postoperatively (T2) | 24.8±7.0 | 30.5±7.3 | 33.2±7.5 | 37.1±7.3 |
| 1 month postoperatively (T3) | 22.1±7.1 | 26.3±7.0 | 28.1±6.5 | 32.8±7.0 |
| 1 year postoperatively (T4) | 11.8±3.7 | 16.6±3.8 | 18.5±3.8 | 21.5±3.8 |

## **Supplementary Table S2. Linear mixed-effects model for Visual Analog Scale (VAS) scores**

| Variable | | β | 95% CI | P value |
| --- | --- | --- | --- | --- |
| Intercept | | 7.148 | 7.048, 7.248 | <0.01 |
| Vitamin Status Group | Insufficiency vs. sufficiency | 0.081 | −0.021, 0.182 | 0.12 |
|  | Deficiency vs. sufficiency | 0.512 | 0.407, 0.616 | <0.01 |
|  | Severe deficiency vs. sufficiency | 0.814 | 0.694, 0.934 | <0.01 |
| VAS | Day 1 postoperatively vs. 1 day before surgery | −4.187 | −4.294, −4.080 | <0.01 |
|  | 1 month postoperatively vs. 1 day before surgery | −5.486 | −5.593, −5.379 | <0.01 |
|  | 1 year postoperatively vs. 1 day before surgery | −6.164 | −6.271, −6.057 | <0.01 |

## **Supplementary Table S3. Linear mixed-effects model for Oswestry Disability Index (ODI) scores**

| Variable | | β | 95% CI | P value |
| --- | --- | --- | --- | --- |
| Intercept | | 63.931 | 63.215, 64.647 | <0.01 |
| Vitamin Status Group | Insufficiency vs. sufficiency | 3.704 | 2.978, 4.429 | <0.01 |
|  | Deficiency vs. sufficiency | 4.998 | 4.248, 5.748 | <0.01 |
|  | Severe deficiency vs. sufficiency | 8.112 | 7.253, 8.972 | <0.01 |
| ODI | Day 1 postoperatively vs. 1 day before surgery | −36.859 | −37.623, −36.095 | <0.01 |
|  | 1 month postoperatively vs. 1 day before surgery | −40.966 | −41.730, −40.201 | <0.01 |
|  | 1 year postoperatively vs. 1 day before surgery | −51.079 | −51.843, −50.315 | <0.01 |
